# Health impact of using anti-PD-(L)1 agents to treat early-stage cancers in Switzerland: a modeling study

**DOI:** 10.3389/fimmu.2025.1601377

**Published:** 2025-07-03

**Authors:** Andrea Favre-Bulle, Maja Stanković, Tyler Mantaian, Claudia Frei, Sonja Schaefer, Sarah Sharon Gabriel, Demet Sönmez, Raquel Aguiar-Ibáñez

**Affiliations:** ^1^ Global Human Health, MSD, Lucerne, Switzerland; ^2^ Lumanity, Bethesda, MD, United States; ^3^ Global Medical and Scientific Affairs, MSD, Lucerne, Switzerland; ^4^ European Clinical Development, Oncology, MSD, Zurich, Switzerland; ^5^ Global Medical and Scientific Affairs, MSD, Stockholm, Sweden; ^6^ Merck Canada, Inc., Kirkland, QC, Canada

**Keywords:** immune checkpoint inhibitors, melanoma, nivolumab, pembrolizumab, renal cell carcinoma, triple-negative breast cancer

## Abstract

**Background:**

Inhibitors of programmed cell death protein 1 (PD-1) and its ligand (PD-L1) (referred to hereafter as anti-PD-(L)1 agents) are approved to treat a variety of advanced-stage cancers. Incorporating these agents into neoadjuvant/adjuvant treatment regimens for early-stage cancers may provide health and economic benefits at the population level.

**Methods:**

A health outcomes projection model compared two scenarios in Switzerland: I) anti-PD-(L)1 agents used only for advanced/metastatic disease, and II) anti-PD-(L)1 agents starting in the neoadjuvant/adjuvant setting. The model focused on three cancers for which anti-PD-(L)1 agents are currently approved in Europe in early stages: melanoma, renal cell carcinoma (RCC), and triple-negative breast cancer (TNBC), projecting clinical evolution over 10 years. Estimated outcomes included life-years, quality-adjusted life-years (QALYs), recurrences/events, active treatments for metastatic disease, adverse events, and deaths.

**Results:**

Of the estimated 10,659 eligible patients during 2022-2031, 9,050 were predicted to initiate neoadjuvant and/or adjuvant treatment with anti-PD-(L)1 agents for treatment of melanoma, RCC, or TNBC. Compared to anti-PD-(L)1 agents being available only in the metastatic setting, use of anti-PD-(L)1 agents in the neoadjuvant and/or adjuvant setting for these 3 cancers was projected to avoid 1,144 recurrences (a 27% decrease), prevent 1,577 active treatments in the metastatic setting (a 35% decrease), avoid 530 deaths (a 23% decrease), and increase life-years without recurrence by 3,416 (a 10% increase).

**Conclusion:**

The use of anti-PD(L)1 agents to treat early-stage cancers in Switzerland is anticipated to result in better outcomes by preventing recurrences/events, active metastatic treatments, and deaths.

## 1 Introduction

Early detection and treatment can reduce the burden of cancer, which is a major health issue in Europe (1–3). It is estimated that 45,000 new cases of cancer and 17,300 deaths due to cancer occur annually in Switzerland (4). Incidence of breast cancer and melanoma is on the rise in Switzerland (5), which makes early detection and treatment even more important. Renal cell carcinoma (RCC) is one of the most common cancers in Switzerland, and in 80% of the cases, it is diagnosed at an early stage (4, 6). It has been projected that, between 2018 and 2040, there will be 98,000 premature cancer deaths in Switzerland, with a productivity cost of €450,000 per death (7).

Immune checkpoint inhibitors (ICIs), primarily inhibitors of programmed cell death protein 1 (PD-1) or its ligand, programmed cell death ligand 1 (PD-L1), and cytotoxic T-lymphocyte-associated protein 4 (CTLA-4), are now a standard treatment for many types of advanced/metastatic cancer (8). ICIs have the potential of benefiting patients with early-stage cancers (9–11), for whom there is still a high unmet need with the standard of care, as recurrence rates remain high (12–16). The question of expanding these agents as neoadjuvant (before surgery to shrink the tumor) or adjuvant (after surgery to prevent or delay recurrence) treatment options for early-stage tumors requires weighing the higher upfront treatment costs against long-term disease-free survival and other potential benefits.

A health outcomes model was developed to assess the impact of adopting PD-1 or PD-L1 inhibitors (referred to hereafter as anti-PD-(L)1 agents) for the neoadjuvant/adjuvant treatment of early-stage cancers versus reserving them for advanced/metastatic disease (17). In this study, the model was used to determine the health impact of using anti-PD-(L)1 agents in 3 different early-stage cancers in Switzerland over a 10-year period: melanoma, RCC, and triple-negative breast cancer (TNBC).

## 2 Materials and methods

### 2.1 Description of the model

The model was designed to quantify the health impact of adding anti-PD-(L)1 agents to traditional cancer treatment/management strategies in the neoadjuvant/adjuvant setting. It compares the difference in clinical outcomes between a scenario in which anti-PD-(L)1 agents are reserved for the advanced/metastatic setting (scenario I) and a scenario in which anti-PD-(L)1 agents are added to the traditional neoadjuvant/adjuvant chemotherapy options (scenario II). Patients enter the model in weekly cycles and initiate treatment with either an anti-PD-(L)1 agent or traditional treatment/management strategies in the neoadjuvant/adjuvant setting. The model tracks the clinical outcomes throughout the average patient journey from initial treatment to recurrence, metastasis, and/or death. Clinical outcomes for the average patient are scaled to account for the number of patients initiating each adjuvant treatment each week throughout the model time horizon. The difference in clinical outcomes between scenario I and scenario II quantifies the impact of adding anti-PD-(L)1 agents to the neoadjuvant/adjuvant treatment/management options.

### 2.2 Base case settings

The analysis was done from the healthcare system perspective and focused on 3 cancer types: melanoma, RCC, and TNBC. **Table 1** shows the base case settings of the model for both scenarios.

**Table 1:** Base case settings.

Input	Value/description
Perspective	Swiss healthcare system
Time horizon	10 years (2022-2031)
Discounting for clinical outcomes^A^	1.50%
Tumor sites	• Stage III melanoma • RCC at increased risk of recurrence following nephrectomy +/- resection of metastatic lesions • Locally advanced or early-stage TNBC at high risk of recurrence
Population	• Population specific for each tumor (melanoma, RCC and TNBC), based on the reported incidence 1990–2019 for each tumor (21), projected growth rate and estimated patient pathways for specific indications. • Females: 50.37% (59).
Health state transitions	Markov model. Transition probabilities are informed from clinical trials and subsequent network meta-analysis or published research.
Treatment duration	Specific to the treatment options received in neoadjuvant/adjuvant setting or in 1L and 2L treatment.
Market shares	Based on market research and expert opinion.
Retreatment with anti-PD-(L)1 agents	Retreatment with any or the same anti-PD-(L)1 agent is assumed 18 months after the neoadjuvant/adjuvant treatment initiation.
Adverse events	Drug-related, grade 3+ with ≥5% incidence in any treatment arm. Adverse events and corresponding disutilities are accounted for as one-time events at treatment initiation.
Health state utilities	Informed from relevant clinical trials and mapped to local values using European algorithms. Age and sex related disutilities are also considered.

1L, first-line; 2L, second-line; PD-(L)1, programmed cell death protein 1 and its ligand; RCC, renal cell carcinoma; TNBC, triple-negative breast cancer.

^A^Discount rate was chosen per the Belgian methodological guidelines for pharmacoeconomic evaluations, in the absence of Swiss specific guidance (60).

### 2.3 Inputs and data sources

#### 2.3.1 Population

The target population was patients with melanoma, RCC, and TNBC in Switzerland who were eligible for neoadjuvant/adjuvant treatment with anti-PD-(L)1 agents. Eligibility for anti-PD-(L)1 agents among cancer patients in Switzerland was determined based on the inclusion criteria from the pivotal trials of pembrolizumab in melanoma (KEYNOTE 054 (18)), early-stage RCC (KEYNOTE 564 (19)), and TNBC (KEYNOTE 522 (20)). Briefly, this included patients with stage III melanoma who underwent complete resection; RCC patients with increased risk of recurrence following nephrectomy, or following nephrectomy and resection of metastatic lesions; and TNBC patients with locally advanced or early-stage cancer at high risk of recurrence (**Table 1**). The number of patients with melanoma, RCC, and TNBC in Switzerland in 2022 was obtained from the Krebsliga Report (4). A combination of scientific literature, internal/external quantitative market research, and consultation with scientific leaders helped to estimate the proportions of the population eligible for treatment with anti-PD-(L)1 agents in the early-stage setting. These numbers were projected out to 2031, assuming the same growth rate as shown in the national incidence reports from 1990-2019 (21).

#### 2.3.2 Treatment patterns

In scenario I, 100% of patients were allocated to management strategies currently used in the early-stage setting. For melanoma, this consisted of watchful waiting or dabrafenib + trametinib; for RCC, it was watchful waiting; and for TNBC, it was chemotherapy. In scenario II, increasing proportions of the patient population were allocated to anti-PD-(L)1 agents as a class over time, using market shares projected from internal estimates. Anti-PD-(L)1 agents were assumed to enter the treatment landscape immediately in the base case. In the current iteration, the model included pembrolizumab and nivolumab, 2 anti-PD-(L)1 agents currently approved for neoadjuvant/adjuvant treatment of cancer in Europe (22, 23).

First-line (1L) and second-line (2L) metastatic therapy was conditional on the treatment received in the adjuvant/neoadjuvant setting and was based on published cost-effectiveness analyses (for melanoma and TNBC (24, 25)) and internal market research (for RCC). Duration of treatment in the base case analysis was based on the Kaplan-Meier curves for time to treatment discontinuation from the pivotal trials (18–20); or on the parametric distributions of patients in each health state (described in the next section); or on the assumption of equivalence to the progression-free survival time (also described below). For all 3 cancers, retreatment with any or the same anti-PD-(L)1 agent was assumed to be 1.5 years after initiation of adjuvant/neoadjuvant treatment (in the absence of local guidelines).

#### 2.3.3 Transitions between health states

The transitions between health states were aligned with those used in prior cost-effectiveness and budget impact models (24, 26–28) accepted by health technology assessment agencies such as the United Kingdom’s National Institute for Health and Care Excellence (29–31). Generally, the population of the recurrence-/event-/disease-free state was modeled as parametric distributions fitted to patient-level data from key pivotal trials (i.e., dependent on the adjuvant/neoadjuvant treatment arm) (18–20), and then used to estimate the probability of transitioning to the locoregional recurrence, metastatic, and death states. Transition probabilities to death were defined as either the trial-based mortality estimates or the background mortality, whichever was larger.

For melanoma and RCC, transitions from the locoregional recurrence state to the metastatic state were assumed to be equivalent across adjuvant treatment arms. These transitions were based on survival analyses of individual patient-level data from database studies (cited in Lai et al. (27) and Favre-Bulle et al. (24)) or were assumed to be equivalent across anti-PD(L)1 agents (18). Transitions to death were assumed to be equivalent across adjuvant treatment arms; in the absence of direct transitions to death or due to the small sample size in the database studies, the maximum between the transition from recurrence-/disease-free to death from the pivotal trial’s intervention arm and the background mortality estimate was used. For TNBC, parametric distributions were fitted to patient-level data; the distribution of the transition from the locoregional recurrence state to the metastatic state or death was based on the pivotal trial data (20).

From the metastatic state to death, several methods were used to derive the transitions for 1L metastatic treatments. These ranged from parametric models (derived from trial data in the advanced/metastatic setting for melanoma and RCC and trial data in the metastatic setting for TNBC) to assuming equivalence between treatments. The parametric models were fitted to patient-level data or based on observed median data, with hazard ratios (HRs) for the remaining treatments derived from network meta-analyses when available (cited in previous studies (24, 26–28)). The transitions for the 1L metastatic treatments were then weighted based on their use, conditional on the adjuvant treatment received, to derive the transitions from the metastatic state that ultimately applied to each adjuvant treatment arm.

Utilities associated with each health state are shown in **Supplementary Table 1** and were derived from key pivotal trials (18–20). Utilities were also applied for age and sex as described previously (32).

#### 2.3.4 Adverse events

Adverse events were assumed to occur with each line of therapy. They were modeled as one-time events, with disutility incurred at treatment initiation. For the adjuvant/neoadjuvant treatment setting, baseline risk and mean duration data for anti-PD-(L)1 agents (average weeks per event multiplied by the number of events per patient) were taken from key trials for melanoma (18), RCC (19), and TNBC (20). In some cases, equivalence was assumed across anti-PD(L)1 agents. For the 1L and 2L metastatic treatment settings, baseline risk and mean duration data were derived from clinical trials and other publications for melanoma (33–40), RCC (41–47), and TNBC (48–51) (with equivalence between treatments and/or lines of therapy assumed where no relevant data were available). Only grade 2+ diarrhea and all grade 3+ adverse event types (which include rash, diarrhea, increased ALT/AST) with a frequency of ≥5% in any treatment arm were considered. Disutility values for adverse events in the adjuvant/neoadjuvant and metastatic settings were, respectively: -0.05 and -0.13/-0.14 (1L/2L) for melanoma (18, 33, 52); -0.06 and -0.05 for RCC (19, 41); and -0.02 and -0.03 for TNBC (20, 48).

### 2.4 Outputs

Outcomes of the analysis were total life-years, recurrence-/event-/disease-free life-years, quality-adjusted life-years (QALYs), events or recurrences (total number), number of active treatments administered for metastatic disease, adverse events (total number), total deaths, and deaths after the first event or recurrence (**Supplementary Table 2**). Life-years, recurrences, and deaths were linked to specific health states, while treatment for metastatic disease and adverse events were linked to specific treatment settings.

### 2.5 Sensitivity analyses

The impact of uncertainty on the estimated outcomes was assessed in sensitivity analyses. Scenarios of interest included: assuming 100% uptake of anti-PD-(L)1 agents (vs. the base case percentages), 20% increase/decrease of target population for all years, delayed time to launch of anti-PD-(L)1 agents in scenario II, and no allowance of retreatment with anti-PD-(L)1 agents (vs. retreatment allowed in the base case). Inclusion of subgroups of cancer types (melanoma only, RCC only, TNBC only, melanoma + RCC, melanoma + TNBC, and RCC + TNBC; a total of 6 combinations) was assessed in supplementary analyses.

One-way sensitivity analysis was further used to investigate which of the selected variables had the largest effect on the outcomes. The variables tested were general population size in each year, target population characteristics for each cancer type (size of the eligible population, percentage female, age, and weight), incidence of individual cancers (total incidence and fractional incidence of patients meeting the indication criteria for treatment with anti-PD-(L)1 agents), HRs in the metastatic setting for overall and progression-free survival, the utility associated with specific health states, and the disutility associated with adverse events. All input variables were adjusted by +/- 10% and HRs were adjusted to their lower and upper bounds according to their 95% confidence interval.

## 3 Results

### 3.1 Cumulative results

**Table 2** shows the annual and cumulative target population for the analysis, which consisted of all patients in Switzerland eligible to initiate adjuvant/neoadjuvant treatment for melanoma, RCC, or TNBC. Of the 10,659 patients in this group over the study period, 9,050 were estimated to receive treatment for their cancer with anti-PD-(L)1 agents in scenario II.

**Table 2:** Target population and number of patients treated with anti-PD-(L)1 agents, by year.

Sample	2022	2023	2024	2025	2026	2027	2028	2029	2030	2031	Cumulative^B^
Target population^A^	978	1,001	1,021	1,042	1,062	1,082	1,103	1,123	1,123	1,123	10,659
Number of patients receiving anti-PD-(L)1 agents under scenario II	619	776	877	917	939	959	977	996	996	996	9,050

PD-(L)1, programmed cell death protein 1 or its ligand

^A^Target population is the total number of patients in Switzerland with melanoma, RCC, or TNBC who are eligible for treatment with anti-PD-(L)1 agents in the early-stage setting.^B^Values are rounded to the nearest whole number; the cumulative total reflects the unrounded sum.

Comparison of scenarios I and II showed the cumulative health impact of early treatment with anti-PD-(L)1 agents over the study period (**Table 3**, **Supplementary Figure 1**). Using anti-PD-(L)1 agents in early-stage cancers vs. limiting their use to the advanced/metastatic setting resulted in gains in total life-years (+1,280; a gain of 3%), recurrence-/event-/disease-free life-years (+3,416; +10%), and QALYs (+1,422; +4%). Benefits were also estimated in terms of 1,144 fewer recurrences (-27%), 1,577 fewer treatments for metastatic disease (-35%), 109 fewer adverse events (-1%), and 530 fewer deaths (-23%).

**Table 3:** Cumulative base case results.

Health outcome	Scenario I: Anti-PD-(L)1 agents used only for advanced stages	Scenario II: Anti-PD-(L)1 agents used in early stages	Difference (% change)
Life-years, recurrence-/event-/disease-free	34,755	38,171	3,416 (+10%)
Life-years, total	42,443	43,722	1,280 (+3%)
QALYs	35,616	37,038	1,422 (+4%)
Events or recurrences	4,292	3,149	-1,144 (-27%)
Active treatments for metastatic disease	4,555	2,978	-1,577 (-35%)
Adverse events	8,606	8,497	-109 (-1%)
Deaths, total	2,301	1,772	-530 (-23%)
Deaths after first event or recurrence	2,040	1,507	-533 (-26%)

PD-(L)1, programmed cell death protein 1 or its ligand; QALY, quality-adjusted life-year

The numbers indicate the cumulative results obtained for the whole target population under scenarios I and II. The difference column is the mathematical difference between the results for scenario II and scenario I, with the percentage change also given.

### 3.2 Health impact over time

**Figure 1:**
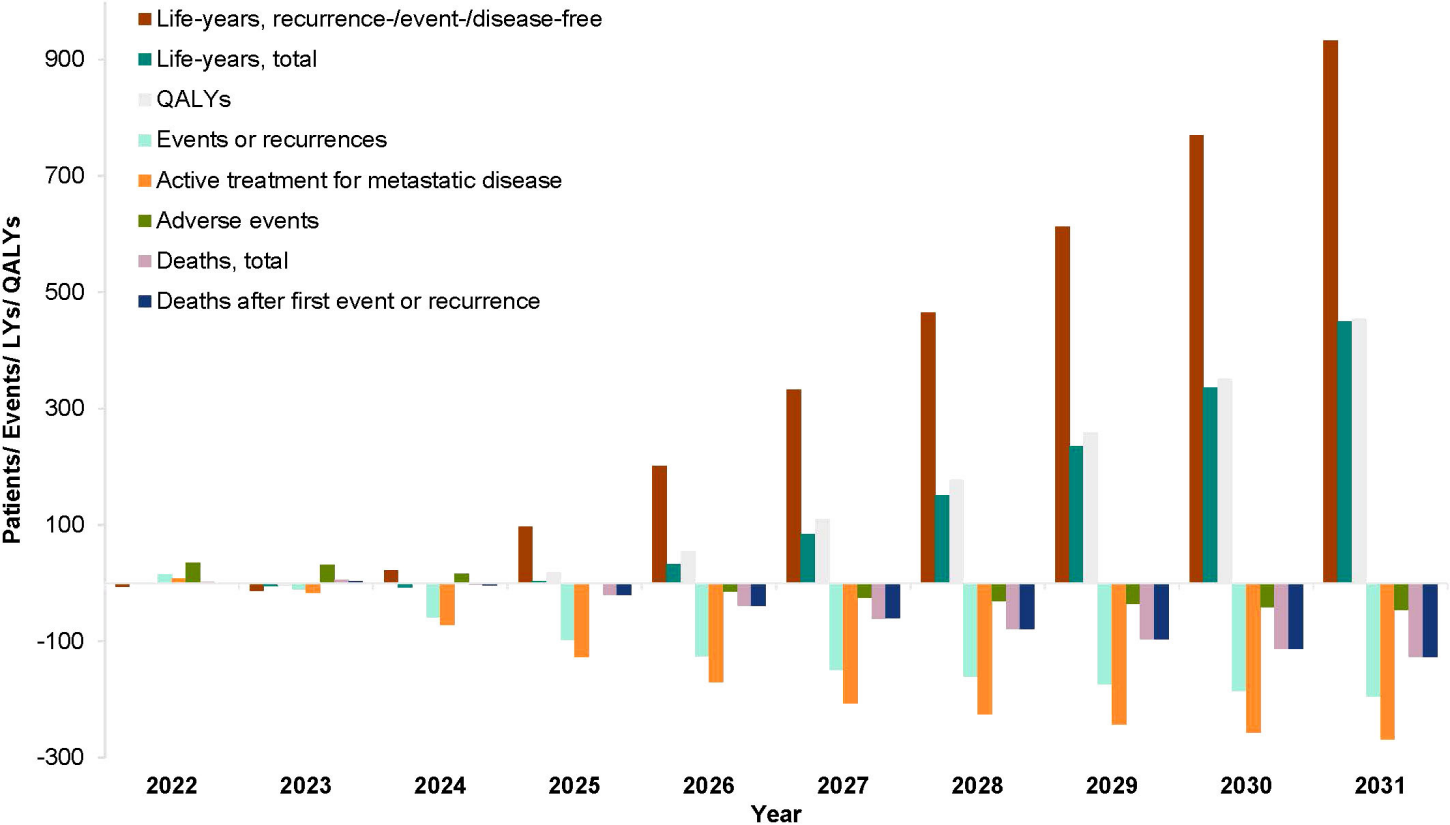
Public health impact of anti-PD-(L)1 agents, by year. QALY, quality-adjusted life-year. The bars indicate the difference between scenarios I and II for the health outcomes listed: life-years (recurrence-/event-/disease-free and total), QALYs, events or recurrences, the number of active treatments for metastatic disease, adverse events, and deaths (after first event/recurrence and total).

**Figure 1** shows the model results for each year of the study period. These data indicate that the gains in life-years and reductions in recurrences and deaths produced by using anti-PD-(L)1 agents in early-stage cancers are not realized until several years after implementation of the scenario II treatment policy. Notably, adverse events increased in the first 3 years and decreased thereafter, for a cumulative decrease of 109 events over the total 10-year period. This is explained by the observation of higher rates of adverse events with anti-PD-(L)1 agents vs. other agents in the adjuvant/neoadjuvant setting, which is eventually offset by the reduction in the number metastatic treatments administered after anti-PD-(L)1 agents, and thus fewer patients experience adverse events in the metastatic setting, resulting in fewer adverse events overall.

### 3.3 Sensitivity analyses

For scenario-based sensitivity analyses, we varied the uptake of anti-PD-(L)1 agents, the target population, the time to launch of anti-PD-(L)1 agents, the capacity for retreatment with anti-PD-(L)1 agents, and the inclusion of various subgroups of cancer. As expected, greater uptake of anti-PD-(L)1 agents led to positive changes in all outcomes (range +13% to +50%), while delayed time to launch of anti-PD-(L)1 agents led to negative changes in all outcomes (range -6% to -60%; **Table 4**). Prohibiting retreatment with anti-PD-(L)1 agents decreased life-years (-6%), QALYs (-2%), deaths (-5%), the number of active treatments for metastatic disease (-10%), and the number of adverse events (-69%). Altering the target population by 20% either way produced numerically equivalent changes in the outcomes (data not shown). Results for selected outcomes and additional scenarios are shown in graphical form in **Supplementary Figure 2**.

**Table 4:** Sensitivity analyses.

Clinical outcomes	Impact on clinical outcomes – Base case scenario	100% uptake of anti-PD-(L)1 agents in future environment	Delayed time to launch of anti-PD-(L)1 agents in future environment	No retreatment with anti-PD-(L)1 agents
Life-years	1,280	+31%	-24%	-6%
QALYs	1,422	+27%	-21%	-2%
Number of events or recurrences	-1,144	+19%	-12%	0%
Number of active treatments for metastatic disease	-1,577	+13%	-6%	-10%
Number of adverse events	-109	+50%	-60%	-69%
Number of deaths	-530	+22%	-15%	-5%
Number of deaths after first event or recurrence	-533	+24%	-15%	-5%

PD-(L)1, programmed cell death protein 1 or its ligand; QALY, quality-adjusted life-year

The percentages indicate the change in each outcome relative to the base case result when the change listed in the column title was applied in scenario II. Plus signs indicate positive changes (either a more positive outcome or a less negative one), and minus signs indicate changes for the worse.

In the one-way sensitivity analysis (data not shown), the variables with the greatest effect on the results were target population size in years 2022–2025 and the HR for overall survival in melanoma and RCC with pembrolizumab vs. other agents or observation. However, the variation in results was minimal, and values were similar to the base case values for all outcomes of interest.

## 4 Discussion

We modeled the health impact of making anti-PD-(L)1 agents available for the treatment of early-stage cancer vs. reserving them for the advanced/metastatic setting in Switzerland. The analysis included 3 cancers for which anti-PD-(L)1 agents are indicated for early-stage treatment in Europe. Our results support the incorporation of anti-PD-(L)1 agents into early-stage cancer treatment based on the expected reductions in deaths, recurrences, metastatic treatments, and adverse events at the national level.

Recent studies demonstrate the rapid uptake of ICIs for advanced cancers in Switzerland. Among 41 patients with unresectable advanced/metastatic BRAF mutation-positive cutaneous melanoma treated at one of 5 hospitals in 2016-2018, ICIs were the most common 1L treatment (70.7% of regimens) and the most common treatment overall (52.9% across multiple lines of therapy) (53). Similarly, among 93 stage III/IV melanoma patients receiving adjuvant therapy after tumor resection at Bern University Hospital’s Cancer Center, 96.8% received ICIs (either pembrolizumab or nivolumab) (54). In a broader analysis of medical records from the Bern University Hospital Cancer Center including 5,109 patients with all types of cancer, Wahli et al. showed that the percentage of cancer patients prescribed ICIs increased from 8.6% in 2017 to 22.9% in 2021 and that ICIs constituted 13.2% of the anticancer treatments in 2017, and 28.2% in 2021 (55). Notably, 95% of ICI prescriptions were for patients with stage III/IV disease, suggesting an opportunity for expansion of ICIs into early-stage treatment.

Data from the Cancer Registry of Zurich 1980–2015 showed that relative 5-year survival rates have increased significantly for breast cancer (70% in 1980-1989, 89% in 2010-2015) and melanoma (74%-86% in 1980-1989, 94%-96% in 2010-2015) in the past few decades (56). Real-world studies support the role of ICIs in improving survival rates in melanoma patients in Switzerland. Among patients with stage IV metastatic melanoma treated with chemotherapy in 2008-2009 (n=95) or ICIs in 2008-2014 (n=121) at one of 3 Swiss hospitals, 12-month survival rates in the chemotherapy and ICI groups were 38% and 69%, respectively, and the median overall survival times were 7.4 months and 16.7 months (57).

A recent Markov modeling study found pembrolizumab to be cost-effective vs. observation, the standard of care for stage IIB/C melanoma in Switzerland, with an incremental cost-effectiveness ratio of CHF 27,424/QALY (well below the willingness-to-pay threshold of CHF 100,000/QALY) (24). Notably, this study examined cost-effectiveness in the early-stage setting, and although the base case used a lifetime time horizon, cost-effectiveness was maintained over a shorter horizon of 20 years. Another Swiss study evaluating the cost-effectiveness of pembrolizumab for patients with RCC post-nephrectomy reported an incremental cost-effectiveness ratio of CHF 65,299/QALY compared to observation (28). Similarly, in the United States, adjuvant pembrolizumab for post-nephrectomy RCC was shown to be cost-effective vs. routine surveillance, with an incremental cost-effectiveness ratio of USD 46,327/QALY (27). In TNBC, a Swiss Markov model evaluating event-free survival in the female population found an incremental cost-effectiveness ratio of CHF 14,114/QALY for neoadjuvant/adjuvant pembrolizumab + chemotherapy versus chemotherapy alone, indicating cost-effectiveness (25).

Together, these findings on ICI uptake, survival rates, and cost-effectiveness, along with the results of the current study indicating a significant health benefit, pave the way for the potential use of ICIs in early-stage cancers. Although priority has historically been given to patients with more advanced or aggressive cancers, there is a comparatively greater number of patients with early-stage cancer, and this ultimately translates to more recurrences and deaths at the population level (9). Thus, there is an unmet need to identify effective treatment strategies for early-stage cancers. ICIs as a class, and anti-PD-(L)1 agents in particular, have an important role to play in treatment of early-stage cancers in the neoadjuvant/adjuvant setting (10, 11, 58). At the same time, more work is needed on biomarkers (e.g., immune checkpoint protein expression and tumor mutational burden) as well as prognostic methods to identify the patients most likely to benefit from neoadjuvant/adjuvant treatment.

### 4.1 Limitations

Two limitations must be considered when interpreting the results of this analysis. First, we used a 10-year time horizon. This likely underestimated the full value of anti-PD-(L)1 agents, and a longer time horizon is warranted to fully capture the range of health benefits experienced by patients. Second, the knowledge base regarding new immunotherapies is rapidly evolving, and additional real-world studies of ICIs in melanoma, and especially RCC and TNBC, where there is currently little information, will be needed to support changes in treatment policy and verify their effects.

### 4.2 Conclusions

The use of anti-PD-(L)1 agents in the treatment of early-stage cancers is estimated to result in better health outcomes in Switzerland by reducing the number of recurrences and deaths, extending the time patients spend free of recurrences/events/disease, and reducing the number of treatments for metastatic disease. These findings can help policymakers weigh the benefits of incorporating anti-PD-(L)1 agents into treatment guidelines for early-stage cancer and support discussions around investment in anti-PD-(L)1 agents in the adjuvant/neoadjuvant setting.

## Data Availability

The original contributions presented in the study are included in the article/**Supplementary Material**. Further inquiries can be directed to the corresponding author.
