# Supplementary material for: Health impact of using anti-PD-(L)1 agents to treat early-stage cancers in Switzerland: a modeling study
Source: Front Immunol. 2025 Jul 3;16:1601377. doi: 10.3389/fimmu.2025.1601377 (PMC12267198; doi:10.3389/fimmu.2025.1601377)
Supplement: Supplementary file 1 [file DataSheet1.docx]

Supplementary Material

# Supplementary Figures and Tables

## Supplementary Tables

Supplementary Table 1. Utilities for health states, by cancer

| Cancer | Melanoma | Renal cell carcinoma | Triple-negative breast cancer |
| --- | --- | --- | --- |
| Recurrence-/event-/disease-free | 0.84 | 0.87 | 0.89^A^ |
| Locoregional recurrence | 0.81 | 0.84 | 0.89 |
| Metastatic, pre-progression | 0.77 | 0.80 | 0.83^B^ |
| Metastatic, post-progression | 0.59 | 0.77 | 0.74^B^ |
| Sources | [[18](#_ENREF_18), [60](#_ENREF_60)] | [[19](#_ENREF_19)]^C^ | [[20](#_ENREF_20), [61](#_ENREF_61), [62](#_ENREF_62)] |

^A^ Defined for TNBC as event-free, on or off treatment.

^B^ Utility value is for the metastatic state, regardless of pre-progression or post-progression status.

^C^ To convert EQ-5D-5L responses to utility scores, the European Union tariff was used in Switzerland in the base case.

Supplementary Table 2. Description of model outputs

| Outcome | Difference between world with and world without anti-PD-(L)1 agents (impact within the model time horizon) | Health state to which the outcome is linked | | | | Treatment setting to which the outcome is linked | | |
| --- | --- | --- | --- | --- | --- | --- | --- | --- |
|  |  | Recurrence-/ event-/ disease-free | Locoregional recurrence | Metastatic | Death | Adjuvant | 1L metastatic | 2L metastatic |
| LYs | Number of LYs gained | ✓ | ✓ | ✓ |  |  |  |  |
| QALYs | Number of QALYs gained | ✓ | ✓ | ✓ |  |  |  |  |
| Number of events or recurrences | Number of recurrences avoided |  | ✓ | ✓ |  |  |  |  |
| Number of active treatments for metastatic disease | Number of treatments for advanced disease avoided |  |  |  |  |  | ✓ | ✓ |
| Number of AEs | Number of AEs avoided |  |  |  |  | ✓ | ✓ | ✓ |
| Number of deaths | Number of deaths avoided |  |  |  | ✓ |  |  |  |
| Number of deaths after first event or recurrence | Number of deaths avoided after event or recurrence |  |  |  | ✓ |  |  |  |

Abbreviations: 1L, first-line; 2L, second-line; AE, adverse event; LY, life-year; PD-(L)1, programmed cell death protein 1 or its ligand; QALY, quality-adjusted life-year

## Supplementary Figures

Supplementary Figure 1. Public health impact of anti-PD-(L)1 agents (cumulative over the 2022-2031 period). Bars show the difference between scenario I and scenario II for each health outcome listed. Abbreviations: PD-(L)1, programmed cell death protein 1 or its ligand; QALY, quality-adjusted life-year

Supplementary Figure 2. Sensitivity analyses of selected outcomes with additional scenarios. Public health impact of anti-PD-(L)1 agents (cumulative over the 2022-2031 period). In each graph, the vertical line indicates the base case result. Bars indicate the change from the base case result. Abbreviations: PD-(L)1, programmed cell death protein 1 or its ligand; RCC, renal cell carcinoma; TNBC, triple-negative breast cancer
